# Increased liver stiffness is associated with mortality in HIV/HCV coinfected subjects: The French nationwide ANRS CO13 HEPAVIH cohort study

**DOI:** 10.1371/journal.pone.0211286

**Published:** 2019-01-25

**Authors:** Sarah Shili-Masmoudi, Philippe Sogni, Victor de Ledinghen, Laure Esterle, Marc-Antoine Valantin, Isabelle Poizot-Martin, Anne Simon, Eric Rosenthal, Karine Lacombe, Gilles Pialoux, Olivier Bouchaud, Anne Gervais-Hasenknoff, Cécile Goujard, Lionel Piroth, David Zucman, Stéphanie Dominguez, François Raffi, Laurent Alric, Firouzé Bani-Sadr, Caroline Lascoux-Combe, Daniel Garipuy, Patrick Miailhes, Daniel Vittecoq, Claudine Duvivier, Hugues Aumaître, Didier Neau, Philippe Morlat, François Dabis, Dominique Salmon, Linda Wittkop, for the ANRS CO13 HEPAVIH study group

**Affiliations:** 1 Univ Bordeaux, ISPED, Inserm Bordeaux Population Health, team MORPH3EUS, UMR 1219, CIC-EC 1401, Bordeaux, France; 2 Centre Hospitalier Universitaire de Bordeaux, Hôpital Haut-Lévèque, Service d’Hépatologie, Bordeaux, France; 3 Assistance Publique des Hôpitaux de Paris, Hôpital Cochin, Service d’Hépatologie, Paris, France; 4 INSERM U-1223 –Institut Pasteur, Paris, France; 5 Université Paris Descartes, Paris, France; 6 Univ Bordeaux, Inserm, UMR 1053, Bordeaux, France; 7 Assistance Publique des Hôpitaux de Paris, Hôpital Pitié-Salpétrière, Service Maladies infectieuses et tropicales, Paris, France; 8 Aix Marseille Univ, APHM Sainte-Marguerite, Service d’Immuno-hématologie clinique, Marseille, France; 9 Inserm U912 (SESSTIM) Marseille, France; 10 Assistance Publique des Hôpitaux de Paris, Hôpital Pitié-Salpétrière, Département de Médecine Interne et Immunologie Clinique, Paris, France; 11 Centre Hospitalier Universitaire de Nice, Service de Médecine Interne et Cancérologie, Hôpital l’Archet, Nice, France; 12 Université de Nice-Sophia Antipolis, Nice, France; 13 Assistance Publique des Hôpitaux de Paris, Hôpital Saint-Antoine, Service Maladies infectieuses et tropicales, Paris, France; 14 UMPC (Université Pierre et Marie Curie), UMR S1136, Institut Pierre Louis d'Epidémiologie et de Santé Publique, Paris, France; 15 Assistance Publique des Hôpitaux de Paris, Hôpital Tenon, Service Maladies infectieuses et tropicales, Paris, France; 16 Assistance Publique des Hôpitaux de Paris, Hôpital Avicenne, Service Maladies infectieuses et tropicales, Bobigny, France; 17 Université Paris 13 Nord, Bobigny, France; 18 Assistance Publique des Hôpitaux de Paris, Hôpital Bichat Claude Bernard, Service des maladies infectieuses et tropicales, Paris, France; 19 Assistance Publique des Hôpitaux de Paris, Hôpital Bicêtre, Hôpitaux universitaires Paris Sud, Service Médecine interne et Immunologie clinique, Le Kremlin-Bicêtre, France; 20 Université Paris Sud, Le Kremlin-Bicêtre, France; 21 Centre Hospitalier Universitaire de Dijon, Département d’Infectiologie, Dijon, France; 22 Université de Bourgogne, Dijon, France; 23 Hôpital Foch, unité VIH, Suresnes, France; 24 Assistance Publique des Hôpitaux de Paris, Hôpital Henri Mondor, Service Immunologie clinique et maladies infectieuses, Immunologie clinique, Créteil, France; 25 Centre Hospitalier Universitaire de Nantes, Service Maladies infectieuses et tropicales, Nantes, France; 26 Centre Hospitalier Universitaire de Toulouse, Hôpital Purpan, Médecine interne, Toulouse, France; 27 Université Toulouse III, Paul Sabatier, Toulouse, France; 28 Centre Hospitalier Universitaire de Reims, Service de médecine interne, maladies infectieuses et immunologie clinique, Reims, France; 29 Université de Reims, Champagne-Ardenne, Reims, France; 30 Assistance Publique des Hôpitaux de Paris, Hôpital Saint-Louis, Service Maladies infectieuses et tropicales, Paris, France; 31 Centre Hospitalier Universitaire de Toulouse, Hôpital Purpan, Maladies infectieuses et tropicales, Toulouse, France; 32 Service des Maladies Infectieuses et Tropicales, CHU Lyon, Hôpital de la Croix Rousse, Lyon, France; 33 Assistance Publique des Hôpitaux de Paris, Hôpital Bicêtre, Hôpitaux universitaires Paris Sud, Service Maladies infectieuses et tropicales, Le Kremlin-Bicêtre, France; 34 APHP-Hôpital Necker-Enfants malades, Service de Maladies Infectieuses et Tropicales, Paris, France; 35 Centre d'Infectiologie Necker-Pasteur, Paris, France; 36 Centre Hospitalier de Perpignan, Service Maladies infectieuses et tropicales, Perpignan, France; 37 Centre Hospitalier Universitaire de Bordeaux, Service Maladies infectieuses et tropicales Bordeaux, Hôpital Pellegrin, Bordeaux, France; 38 Centre Hospitalier Universitaire de Bordeaux, Service de médecine interne, hôpital Saint-André, Bordeaux, France; 39 Centre Hospitalier Universitaire de Bordeaux, Pôle de Santé Publique, Bordeaux, France; 40 Assistance Publique des Hôpitaux de Paris, Hôpital Cochin, Service Maladies infectieuses et tropicales, Paris, France; Harvard Medical School, UNITED STATES

## Abstract

**Background:**

The association between liver stiffness measurements (LSM) and mortality has not been fully described. In particular the effect of LSM on all-cause mortality taking sustained virological response (SVR) into account needs further study.

**Methods:**

HIV/HCV participants in the French nation-wide, prospective, multicenter ANRS CO13 HEPAVIH cohort, with ≥1 LSM by FibroScan (FS) and a detectable HCV RNA when the first valid FS was performed were included. Cox proportional hazards models with delayed entry were performed to determine factors associated with all-cause mortality. LSM and SVR were considered as time dependent covariates.

**Results:**

1,062 patients were included from 2005 to 2015 (69.8% men, median age 45.7 years (IQR 42.4–49.1)). 21.7% had baseline LSM >12.5 kPa. Median follow-up was 4.9 years (IQR 3.2–6.1). 727 (68.5%) were ever treated for HCV: 189 of them (26.0%) achieved SVR. 76 deaths were observed (26 liver-related, 10 HIV-related, 29 non-liver-non-HIV-related, 11 of unknown cause). At the age of 50, the mortality rate was 4.5% for patients with LSM ≤12.5 kPa and 10.8% for patients with LSM >12.5 kPa. LSM >12.5 kPa (adjusted Hazard Ratio [aHR] = 3.35 [2.06; 5.45], p<0.0001), history of HCV treatment (aHR = 0.53 [0.32; 0.90], p = 0.01) and smoking (past (aHR = 5.69 [1.56; 20.78]) and current (3.22 [0.93; 11.09]) versus never, p = 0.01) were associated with all-cause mortality independently of SVR, age, sex, alcohol use and metabolic disorders.

**Conclusion:**

Any LSM >12.5 kPa was strongly associated with all-cause mortality independently of SVR and other important covariates. Our results suggest that close follow-up of these patients should remain a priority even after achieving SVR.

## Background

Hepatitis C Virus (HCV) infection is frequent in people living with Human Immunodeficiency Virus (HIV), ranging from 6.2% to 82.4% in injection drug users [1]. HIV/HCV co-infection leads to an increased mortality compared to HIV mono-infected patients [2, 3], and a more rapid liver disease progression [4, 5]. Liver-related mortality has been ranked first [6, 7] with a decline in more recent calendar years [8, 9]. Early administration of combination antiretroviral therapy (cART) and durable suppression of HIV replication have improved overall survival and delayed disease progression [10–12]. Despite cART, liver disease remains currently a leading cause of death [13–15], probably due to hepatic decompensation (14) and/or hepatocellular carcinoma [15]. In the era of direct acting antivirals (DAAs), sustained virological response (SVR) ranges from 85–100%; these high percentages have been obtained in randomized clinical trials [16, 17] as well as in real-life settings [18, 19], both HCV mono-infected and HIV/HCV co-infected patients. Nonetheless its impact on long-term disease progression needs further study.

There are several studies linking advanced fibrosis stages or cirrhosis including non-invasive markers of fibrosis with a higher risk for all-cause mortality, liver-related mortality or events [13, 20–22]. Other studies assessed factors associated including liver fibrosis stages with mortality or liver-related mortality with a focus on the effect of SVR on disease progression [23–26]. Some of these studies may suffer from methodological issues as they did not consider SVR as a time-dependent covariate, implicating a potential immortal time bias or an indication bias (SVR is directly linked to treatment and its indications). Furthermore, liver fibrosis stages may vary over time and can be influenced by SVR [27, 28]. In recent studies assessing the association between liver fibrosis stages and hepatocellular carcinoma, liver-related events, or end stage liver disease markers of liver fibrosis were not analyzed as time-dependent variables in their adjusted models. Some recent studies did not consider SVR at all [13, 21]. Thus, the association between liver stiffness measurements (LSM, a surrogate marker for liver fibrosis stages [29]) and its association with all-cause and liver-related mortality has not been fully described. In particular the effect of LSM on all-cause mortality taking SVR and its effect on LSM into account needs further study.

We assessed the effect liver stiffness measurements (and its evolution over time) with all-cause and liver-related mortality in HIV/HCV co-infected patients from the prospective French nationwide ANRS CO13 HEPAVIH cohort.

## Patients and methods

### Study population

ANRS CO13 HEPAVIH is a French nationwide multicenter, observational cohort study of HIV/HCV co-infected patients with three inclusion phases: 2005–2008, 2011–2015, 2014–2015 [30]. All patients provided written consent for study participation and the ANRS CO13 HEPAVIH cohort has received approval by an Institutional Review board (CPP Ile de France III, file n°2234, ref CG/LG/CC 2005–255). Each participant agreed to participate to the ANRS CO13 Hepavih cohort by written consent. Data were prospectively collected using standardized case report forms including information on patient demographics, health-related behaviors (drug, tobacco and alcohol use), clinical diagnoses and laboratory tests on a yearly basis, or every six months if patients had cirrhosis. For this analysis, adult patients above 18 years with at least one LSM by FibroScan (FS) meeting the validity criteria (IQR/LSM≤30% and ten measurements, according to current guidelines), a detectable HCV ribonucleic acid (RNA), and at least one follow-up visit before 1^st^ October 2015 (administrative censoring) were included. LSM by FS is a non-invasive and repeatable tool that has been validated in HIV/HCV co-infected patients [29]. Patients entered the study at the date of the first valid FS.

### Clinical outcomes

The primary and secondary outcomes were all-cause mortality and liver-related mortality (death from end stage liver disease, liver cancer or complication of liver transplantation), respectively. Patient’s status was ascertained by declaration of participating centers and by research of their vital status in the national death registry for patients lost-to follow-up >24 months. Causes of death were reviewed by a validation committee. Patients were censored at their last follow-up date. For the secondary outcome, patients who died from a non-liver-related event were censored at their death date.

### Liver stiffness measurement (LSM)

FS was performed by trained operators in the participating centers. We considered only valid FS with cut-offs for LSM as described in the literature ([2.5–7.1], ]7.1–9.5], ]9.5–12.5] and > 12.5 kPa) [31] or with two categories: ≤12.5 kPa versus >12.5 kPa. LSM is evolving and therefore we considered it as a time-dependent covariate. LSM was also considered as a continuous time-dependent covariate in kPa.

### Sustained virological response

SVR was defined as an undetectable HCV RNA assessed by polymerase chain reaction (PCR) six months after the end of treatment as this analysis included mainly patients treated by peg-interferon/ribavirin. We created a three-modalities variable: untreated (patients never receiving any HCV treatment), treated-SVR-negative (patients under HCV treatment and up to six months after the end of treatment), treated-SVR-positive (patients who achieved SVR six months after the end of their HCV treatment). When HCV RNA six months after treatment was detectable, patients were considered in the treated-SVR-negative category until the achievement of SVR with another HCV treatment. SVR was considered as a time-dependent covariate.

### Others covariates

Current and past alcohol consumption was assessed according to the patients’ declaration to their physician, and classified as follows: never, past non-excessive, past excessive, current non-excessive and current excessive. “Excessive” was defined as >21 glasses / week of alcohol for men, >14 for women according to the World Health Organization. Tobacco and drug use were categorized as never, past or current. Mode of HIV transmission, presence of cART, Acquire immune deficiency syndrome (AIDS) stage and previous HCV treatment by peg-interferon/ribavirin before inclusion (pretreated versus naive) were also assessed from medical records. Laboratory assessments included HCV RNA, HIV RNA and CD4+ cell count as a time-dependent covariates, HCV genotype, hepatitis B serology, and serum chemistry panel. In order to avoid underestimation of metabolic disorders in HIV/HCV co-infected patients, a broad definition was used: presence of at least one of the following conditions: 1/ diabetes or antidiabetic treatment, 2/ lipodystrophy, 3/ insulin resistance with a HOMA-index > 3.8 (34,35), 4/ metabolic syndrome [32].

### Statistical analysis

Survival analysis was conducted to determine factors associated with all-cause mortality and liver-related mortality. The time axis was age allowing for a precise adjustment on age, and being clinically more relevant than time since first valid FS (not corresponding to a particular event for the patient). Survival curves were constructed and compared across initial LSM categories (by Kaplan-Meier method and log-rank test for the primary outcome; Aalen-Johansen method and Gray test for the secondary outcome, to take into account competitive risks).

Cox proportional hazards models with delayed entry were used to study factors associated with mortality. In univariable analysis, factors were selected with a p-value <0.25. In multivariable analysis, SVR, alcohol use, metabolic disorders, sex and LSM were forced into the model and factors associated with mortality (p-value <0.05) were determined with a backward stepwise selection procedure among: 1/ fixed variables determined at inclusion: tobacco use, drug use, mode of HIV transmission, AIDS stage, previous HCV treatment, HCV genotype, HBs antigen; 2/ time-dependent variables: HIV RNA, CD4+ level. HCV RNA and cART were not included in the model due to collinearity with SVR and HIV RNA, respectively. Results were based on patients without missing data. For the analysis of liver-related mortality, the same strategy was applied but the model could not be adjusted for SVR as no patient who achieved SVR died from liver-related cause during follow-up.

A more comprehensive analysis using a joint model with shared random effects was performed as it allows studying more precisely the association between time-dependent LSM and all-cause mortality [33]. The joint model simultaneously estimated the trajectory of LSM (continuous variable in kPa) for which repeated data were available, and the risk of death, taking into account the link between LSM and occurrence of deaths, and the possible variation of LSM between two measurements. It was adjusted for time-dependent SVR, metabolic disorders, sex, CD4+ level, previous HCV treatment and alcohol use determined at inclusion. The effect of LSM on all-cause mortality was estimated through two parameters: the current value of LSM given by a single FS during follow-up, and the trajectory of LSM between two consecutive FS during follow-up. LSM was ln-transformed to satisfy the Gaussian hypothesis.

Statistical analyses were performed using SAS versions 9.3 and 9.4 (SAS Institute Inc., Cary, North Carolina) and JM package in R to estimate the joint model [34].

## Results

### Study population

**Fig 1:**
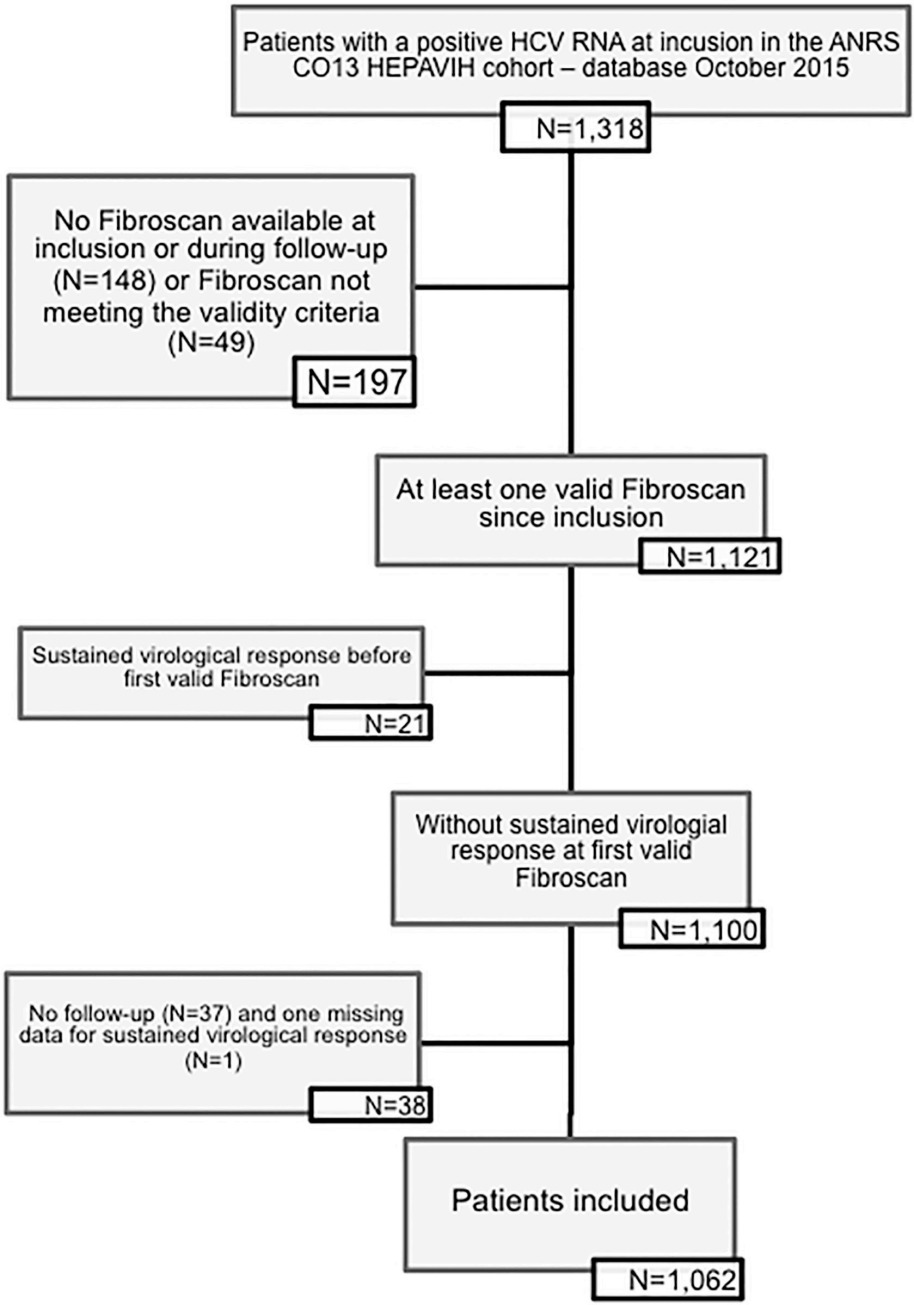
Flowchart.

Of the 1,516 patients included from December 2005 to October 2015, 1,170 had at least one FS since their inclusion in the cohort, and 1,121 met the validity criterion. 1,100 patients met the study inclusion criteria but 38 were excluded for missing data on SVR (one) or absence of follow-up (n = 37). Finally, 1,062 patients were analyzed (Fig 1).

Characteristics of the study population at the time of first valid FS are shown in Table 1. A LSM >12.5 kPa was observed in 21.7% of the patients. In the LSM >12.5 kPa group, patients were older, mostly men, had lower CD4+ counts, had more frequent metabolic disorders, were more often under HCV treatment at inclusion, and were more often pretreated for HCV.

**Table 1:** Characteristics at baseline of HIV/HCV co-infected patients included in this analysis—ANRS CO13 HEPAVIH cohort.

	TOTAL	LSM		
Characteristics	N = 1062	≤12.5 kPa N = 831	>12.5 kPa N = 231	*P*^a^
Age (years), median (IQR)	45.7 (42.4–49.1)	45.3 (42.2–48.8)	47.3 (43.4–51.3)	<0.001
Sex, women, n (%)	321 (30.2)	274 (33.0)	47 (20.4)	<0.001
Alcohol consumption, n (%)				0.008
Never	230 (22.7)	183 (23.1)	47 (21.0)	
past non-excessive	195 (19.2)	138 (17.5)	57 (25.4)	
past excessive	116 (11.4)	84 (10.6)	32 (14.3)	
current non-excessive	384 (37.8)	318 (40.2)	66 (29.5)	
current excessive	90 (8.9)	68 (8.6)	22 (9.8)	
Tobacco consumption, n (%)				0.118
Never	127 (12.1)	106 (12.9)	21 (9.2)	
past	174 (16.6)	128 (15.6)	46 (20.2)	
current	746 (71.3)	585 (71.4)	161 (70.6)	
Drug consumption, n (%)				<0.001
never	279 (26.6)	238 (29.0)	41 (17.9)	
past	711 (67.7)	530 (64.6)	181 (79.0)	
current	60 (5.7)	53 (6.5)	7 (3.1)	
Mode of HIV transmission, n (%)				0.036
heterosexual	38 (4.0)	35 (4.8)	3 (1.4)	
male homosexual	53 (5.6)	45 (6.2)	8 (3.7)	
injection drug user	761 (80.8)	575 (79.0)	186 (86.9)	
other	90 (9.6)	73 (10.0)	17 (7.9)	
Detectable HIV RNA, n (%)	269 (25.3)	216 (26.0)	53 (22.9)	0.346
CD4^+^ cell count(cells/mm^3^), median (IQR)	468.0 (330.0–667.5)	483.0 (350.0–687.0)	408.0 (254.0–598.0)	<0.001
Nadir of CD4+ (cells/mm^3^), median (IQR)	149.0 (69.0–245.5)	160.0 (75.0–256.0)	108.0 (50.0–209.0)	<0.001
Antiretroviral treatment, n (%)	755 (76.3)	599 (76.6)	156 (75.0)	0.630
HIV stage, n (%)				0.170
A (asymptomatic)	187 (28.6)	150 (30.4)	37 (23.0)	
B (symptomatic)	270 (41.3)	196 (39.8)	74 (46.0)	
C (AIDS)	197 (30.1)	147 (29.8)	50 (31.1)	
Time from HCV contamination (years), median (IQR)	21.0 (16.0–25.0)	21.0 (15.0–24.0)	22.0 (18.0–27.0)	0.006
Detectable HCV RNA, n (%)	866 (81.5)	684 (82.3)	182 (78.8)	0.222
Current HCV treatment, n (%)	111 (11.2)	67 (8.6)	44 (21.2)	<0.001
Previous HCV treatment, n (%)	441 (41.5)	306 (36.8)	135 (58.4)	<0.001
HCV genotype, n (%)				0.323
1, 4 or 5	832 (78.9)	646 (78.2)	186 (81.2)	
2 or 3	223 (21.1)	180 (21.8)	43 (18.8)	
Positive HBs antigen, n (%)	22 (2.2)	18 (2.3)	4 (1.9)	1.000
Presence of metabolic disorders, n (%)	609 (57.3)	457 (55.0)	152 (65.8)	0.003
Presence of insulin resistance, n (%)	145 (13.7)	87 (10.5)	58 (25.1)	<0.001
Presence of diabetes, n (%)	36 (3.4)	17 (2.1)	19 (8.2)	<0.001
Presence of metabolic syndrome, n (%)	151 (14.2)	106 (12.8)	45 (19.5)	0.010

IQR, Interquartile Range; HIV, Human Immunodeficiency Virus; HCV, Hepatitis C Virus; AIDS, acquired immunodeficiency syndrome;

^a^Chi-square test or Fisher test for qualitative covariates, Mann-Whitney-Wilcoxon test for quantitative covariates

Median follow-up was 4.9 years (IQR, 3.2–6.1 years). At the end of follow-up, 335 (31.5%) patients had never received an HCV treatment and, among the 727 patients treated at least once, 189 (26.0%) had achieved SVR: 141 (74.6%) after peg-interferon/ribavirin, 38 (20.1%) after telaprevir- or boceprevir-based therapy and 10 (5.3%) after DAA. Among those 189 patients, 11 (5.8%) had a LSM > 12.5 kPa at SVR-time.

### Death risk during follow-up

**Fig 2:**
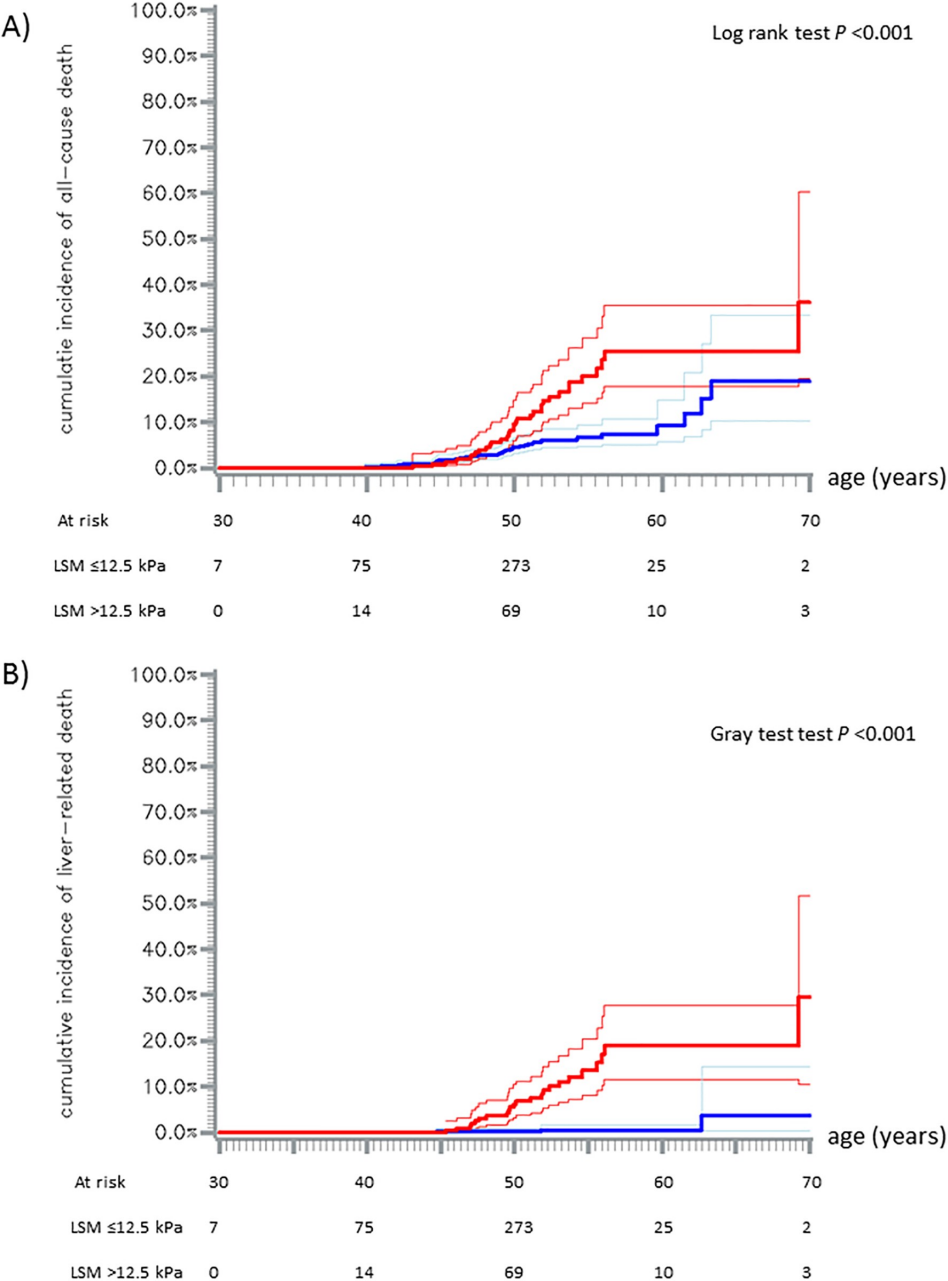
Cumulative incidence of all-cause mortality (A) and liver related mortality (B). Cumulative incidence for patients with LSM ≤ 12.5 kPa (blue line) and patients with LSM >12.5 kPa (red line); 95% confidence intervals are represented by pointed lines. Kaplan-Meier method (A) and Aalen-Johansen method (B).

Overall, we observed 76 deaths (Table 2). No liver transplantation occurred during follow-up. Cumulative incidence of all-cause mortality was 5.9% (95% confidence interval (95% CI): [4.4–7.8]) at the age of 50, 13.4% [10.0–17.9] at the age of 60 and 25.5% [14.7–42.0] at the age of 70. It was higher in the LSM >12.5 kPa group than below this threshold (for example, at 50 years, 10.8% versus 4.5%, log rank test, *p*<0.001) (Fig 2).

**Table 2:** Causes of death—ANRS CO13 HEPAVIH cohort.

Cause of death	n
Overall	76
Liver-related death*:	26 (34.2%)
End stage liver disease**	17
HCC without end stage liver disease	4
Hepatocholangiocarcinoma	1
Liver transplant complications	3
HIV-related death:	10 (13.1%)
Infectious disease	5
Pulmonary hypertension	1
Progressive multifocal leukoencephalopathy	3
Hodgkin lymphoma	1
Non-HIV and non-liver-related death:	29 (38.2%)
Cancer***	12
Infectious disease	2
Psychiatric disease	5
Cardiovascular disease	4
Sudden death	1
Toxic cause	4
Pulmonary fibrosis	1
Undetermined	11 (14.5%)

*One patient was classified as a liver-related but the precise cause was not reported.

**Three patients with end stage liver disease were reported to have hepatocellular carcinoma.

*** 5 broncho-pulmonary, 1 ovarian, 1 esophageal, 1 nasopharyngeal, 1 colon, 2 pancreatic, 1 breast cancers.

Cumulative incidence of liver-related mortality was 1.6% [0.9–2.7] at the age of 50, 5.4% [3.3–8.2] at the age of 60 and 13.6% [4.0–28.9] at the age of 70. It was higher in the LSM >12.5 kPa group than below this threshold (at 50 years, 6.8% versus 0.14%, Gray test, *p*<0.001) (Fig 2).

**Fig 3:**
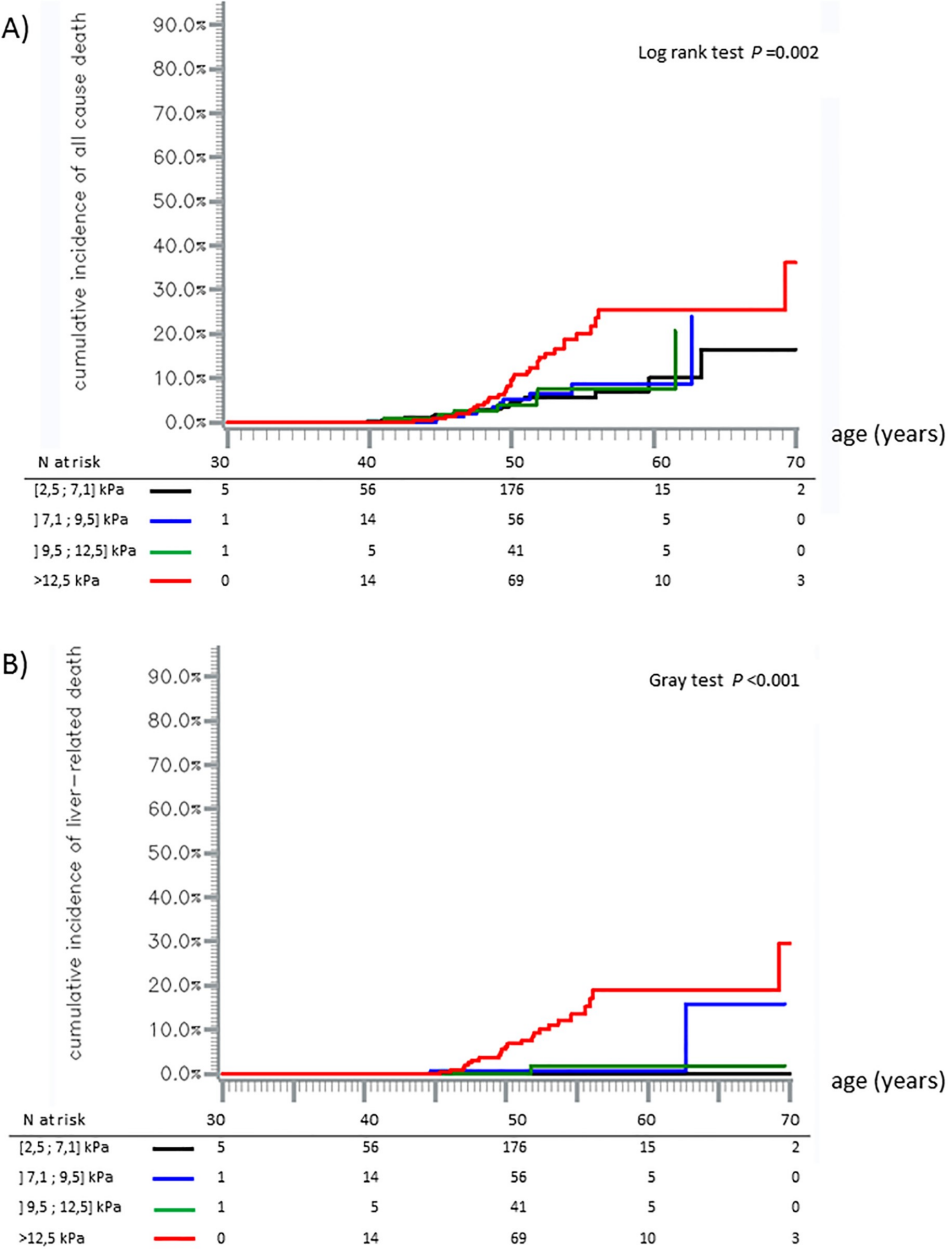
Cumulative incidence of all-cause, mortality (A) and liver-related mortality (B). Black line: LSM [2.5;7.1] kPa, blue line: LSM ]7.1;9.5] kPa, green line: LSM ]9.5;12.5] kPa, and red line: LSM >12.5 kPa. Kaplan-Meier method (A) and Aalen-Johansen method (B).

There was no difference in all-cause mortality nor in liver-related mortality among the three low LSM categories [2.5–7.1],] 7.1–9.5],] 9.5–12.5] (Fig 3).

### LSM and all-cause mortality

In univariable analysis, patients with LSM >12.5 kPa at any point of follow-up had a higher risk of all-cause mortality compared to patients with LSM ≤12.5 kPa (crude HR [cHR] [95% CI] = 3.11 [1.96–4.92], *p*<0.001). All-cause mortality was also more frequent in men, tobacco users and drug users. However, it was lower in ant-HCV pretreated patients and with an increased CD4+ count (Table 3).

**Table 3:** Crude (A) and adjusted (B) hazard ratios from a Cox proportional hazards model with delayed entry for all-cause mortality in HIV/HCV patients—ANRS CO13 HEPAVIH cohort.

	Univariable analysis (A) cHR [95% CI]	*P*	Multivariable analysis (B)* aHR [95% CI]	*P*
LSM (t):		<0.001		<0.001
≤12,5 kPa	1		1	
>12,5 kPa	3.11 [1.96; 4.92]		3.35 [2.06; 5.45]	
SVR (t)				0.002
untreated	1		1	
treated-SVR-	3.12 [1.10; 4.02]		3.53 [1.23; 5.47]	
treated-SVR+	0.62 [0.13; 2.88]		0.75 [0.17;3.81]	
Sex: women *versus* men	0.60 [0.34; 1.04]	0.070	0.71 [0.40; 1.26]	0.243
Alcohol consumption		0.063		0.089
Never	1		1	
past non-excessive	0.75 [0.36; 1.56]		0.58 [0.27; 1.23]	
past excessive	0.40 [0.14; 1.21]		0.31 [0.10; 0.94]	
current non-excessive	0.92 [0.50; 1.68]		0.72 [0.38; 1.38]	
current excessive	1.80 [0.87; 3.73]		1.22 [0.57; 2.62]	
Tobacco consumption		0.048		0.015
never	1		1	
past	4.43 [1.28; 15.33]		5.69 [1.56; 20.78]	
current	2.98 [0.91; 9.74]		3.22 [0.93; 11.09]	
Drug consumption		0.094		0.447
never	1		1	
past	2.10 [1.05; 4.20]		1.53 [0.73; 3.17]	
current	1.30 [0.36; 4.76]		0.99 [0.27; 3.69]	
Mode of HIV transmission		0.704		-
heterosexual	1		-	
male homosexual	1.28 [0.20; 8.03]		-	
injection drug users	1.71 [0.41; 7.17]		-	
others	1.02 [0.19; 5.47]		-	
Detectable HIV viral load (t) versus undetectable HIV viral load (t)	0.73 [0.37; 1.43]	0.360	-	-
CD4+ level (/50 cells/mm^3^) (t)	0.95 [0.90; 0.99]	0.008	1.00 [0.95; 1.00]	0.287
Nadir of CD4+ (/50 cells/mm^3^)	0.96 [0.87; 1.05]	0.341	-	-
HIV stage:		0.950		-
A (asymptomatic)	1		-	
B (symptomatic)	1.02 [0.54; 1.94]		-	
C (AIDS)	1.11 [0.55; 2.21]		-	
HCV genotype		0.852		-
2 or 3 *versus* 1, 4 or 5	0.95 [0.55; 1.64]		-	
Time from HCV contamination (/year)	1.02 [0.97; 1.07]	0.430	-	-
Positive HBs antigen	0.91 [0.13; 6.63]	0.929	-	-
Presence of metabolic disorders	1.04 [0.64; 1.67]	0.886	0.92 [0.56; 1.51]	0.737
Presence of previous anti-HCV treatment	0.60 [0.37; 0.99]	0.046	0.53 [0.32; 0.90]	0.017

cHR, crude Hazard ratio; aHR, adjusted Hazard ratio; LSM, liver stiffness measurements; (t) indicates time-dependent covariables;

*52 patients had missing data on alcohol and tobacco and were not included in the final model. There was no difference between analyzed and excluded patients (data not shown).

In multivariable analysis, LSM >12.5 kPa at any time of follow-up was associated with an increased risk of all-cause mortality (adjusted HR (aHR) = 3.35 [2.06–5.45], p<0.001), independently of SVR, sex, alcohol use, tobacco use, metabolic disorders and previous HCV treatment. In addition, tobacco use (aHR = 5.69 [1.56; 20.78] for past use versus never, 3.22 [0.93; 11.09] for current use versus never, p = 0.015) and previous HCV treatment (aHR = 0.53 [0.32; 0.90], p = 0.017) were also associated with all-cause mortality, independently of SVR and the other covariates (Table 3).

The association of LSM with all-cause mortality was confirmed by the joint model. In the longitudinal sub-model, only SVR was associated with LSM trajectory during follow-up (Table in S1 Table). Furthermore, the model confirmed that an increase in LSM at a given time-point was associated with an increased risk of all-cause mortality, after adjustment for confounders and trajectory of LSM (aHR = 1.64 [1.43–1.88] for a 50% increase of current LSM value, *p*<0.001). The trajectory of LSM between two measurements was not associated with all-cause mortality (Table 4).

**Table 4:** Adjusted hazard ratios for all-cause mortality from the survival sub-model of the joint model with shared random effects in HIV/HCV co-infected patients—ANRS CO13 HEPAVIH cohort.

	aHR [95% CI]*	*P*
Current value of LSM** (for a 50% increase)	1.64 [1.43; 1.88]	<0.001
Trajectory of LSM between the current measure and the previous one** (for a 50% increase)	0.52 [0.01; 56.15]	0.787
SVR (t)		0.006
treated-SVR- *versus* untreated	2.05 [1.15; 3.64]	
treated-SVR+ *versus* untreated	0.41 [0.09; 1.84]	
Sex, women *versus* men	0.91 [0.53; 1.55]	0.731
Alcohol consumption		0.373
past non-excessive *versus* never	0.87 [0.42; 1.81]	
past excessive *versus* never	0.55 [0.21; 1.46]	
current non-excessive *versus* never	1.20 [0.63; 2.30]	
current excessive *versus* never	1.33 [0.62; 2.86]	
CD4^+^ count (/50cells/mm^3^)	0.98 [0.94; 1.03]	0.444
Presence of metabolic disorders	1.11 [0.71; 1.75]	0.645
Presence of previous HCV treatment	0.43 [0.26; 0.72]	0.001

aHR, adjusted Hazard ratio; LSM, liver stiffness measurements; SVR, sustained virological response; (t) indicates time-dependent covariables;

*Model estimations are based on data from 959 patients without missing data for variables included in the model. In this subgroup, 67 all-cause deaths occurred.

**All LSM available during follow-up were considered to calculate the trajectory of LSM, even those obtained after achieving SVR since the model was adjusted for SVR time-dependent covariate. Current value of LSM represented value at a time point t and the trajectory represented the evolution of LSM.

### LSM and liver-related mortality

The risk of liver-related mortality increased when LSM was >12.5 kPa (cHR = 29.65 [8.88–99.01], *p*<0.001), in case of metabolic disorders, and in drug users. It decreased for women, when the CD4+ level increased, for HCV genotype 2 or 3 patients, and for pretreated patients (Table 5).

**Table 5:** Crude (A) and adjusted (B) hazard ratio from a Cox proportional hazards model with delayed entry for occurrence of liver-related death in HIV/HCV co-infected patients from the HEPAVIH cohort.

	Univariable analysis (A) cHR [95% CI]	*P*	Multivariable analysis (B)* aHR [95% CI]	*P*
LSM (t):		<0.001		<0.001
≤12,5 kPa	1		1	
>12,5 kPa	29.65 [8.88; 99.01]		20.60 [5.99; 70.78]	
Sex (women *versus* men)	0.09 [0.01; 0.65]	0.017	0.16 [0.02; 1.22]	0.077
Alcohol consumption**		0.480		0.786
never	1		1	
past non-excessive or excessive	0.52 [0.16; 1.74]		0.52 [0.14; 1.92]	
current non-excessive	0.93 [0.34; 2.57]		0.82 [0.26; 2.62]	
current excessive	1.47 [0.41; 5.26]		0.76 [0.19; 3.07]	
Tobacco consumption**		0.317		-
never	1		-	
past	4.71 [0.56; 39.79]		-	
current	3.13 [0.40; 24.69]		-	
Drug consumption**		0.095		0.570
never	1		1	
past or current	3.52 [0.80; 15.42]		1.56 [0.34; 7.13]	
Mode of HIV transmission		0.546		-
sexual	1		-	
injection drug users	2.70 [0.35; 20.92]		-	
others	1.36 [0.11; 17.19]		-	
Detectable HIV viral load (t)	0.66 [0.20; 2.22]	0.503	-	-
CD4+ count (/50 cells/mm^3^) (t)	0.86 [0.78; 0.95]	<0.001	0.90 [0.82; 1.00]	0.065
Nadir of CD4+ (/50 cells/mm^3^)	0.94 [0.80; 1.11]	0.473	-	-
HIV stage		0.329		-
A (asymptomatic)	1		-	
B (symptomatic)	2.64 [0.74; 9.42]		-	
C (AIDS)	2.15 [0.53; 8.66]		-	
HCV genotype:		0.195		0.243
2 or 3 *versus* 1, 4 or 5	0.95 [0.55; 1.64]		0.47 [0.13; 1.67]	
Time from HCV contamination	1.00 [0.92; 1.09]	0.930	-	-
Presence of metabolic disorders	4.50 [1.35; 15.00]	0.014	3.91 [1.12; 13.66]	0.033
Presence of previous HCV treatment	0.38 [0.15; 0.94]	0.036	0.31 [0.12; 0.85]	0.017

cHR, crude Hazard ratio; aHR, adjusted Hazard ratio; LSM, liver stiffness measurements; HIV, Human immunodefiency virus; HCV, hepatitis C virus; AIDS, acquired immunodeficiency syndrome;

*47 patients had missing data on alcohol and CD4+ count and were not included in the final model. (t) indicates time-dependent covariables. There was no difference between analyzed and excluded patients (data not shown).

**Definition modified for the secondary outcome analysis because no event occurred in some categories of the original variables making the estimation of HRs impossible for these categories.

LSM >12.5 kPa at any time remained associated with an increased risk of liver-related mortality (aHR = 20.60 [5.99–70.78], *p*<0,001) after adjustment for sex, alcohol use, metabolic disorders, CD4+ level and previous HCV treatment. This result was not adjusted for SVR as no liver-related death occurred in the SVR group. Previous HCV treatment (aHR = 0.31 [0.12–0.85], *p* = 0.022) and metabolic disorders (aHR = 3.91 [1.12–13.66], *p* = 0.033) were also associated with liver-related mortality, after adjustment for the other variables (Table 5).

## Discussion

In this large-scale assessment of mortality in a prospective, multicenter, observational cohort study of HIV/HCV co-infected patients, a LSM >12.5 kPa at any time point during follow-up was associated with a 3 fold higher risk of all-cause mortality, independently of SVR and other main confounders. This association has been confirmed by joint modeling of the LSM trajectory and all-cause mortality. Furthermore, the latter analysis showed that mortality was related to an increase in the current LSM value at a given time point, and not to the trajectory of LSM. LSM was also associated with liver-related mortality after adjustment for main confounders. Nevertheless, small number of liver-related deaths hampered the adjustment for SVR and led to large confidence intervals.

Our results on LSM are concordant with previous studies [13, 20, 21, 35]. Limketkai et al found that patients with liver fibrosis stage F4 had a three fold higher risk of death than F0 patients [20]. Sanmartin et al also found a 3.7 fold higher risk of death for patients with advanced fibrosis (F3-F4) [35]. This is probably due to a greater incidence of liver-related events (liver decompensation, hepatocellular carcinoma…) in case of advanced fibrosis (25). Nonetheless, these studies did not consider SVR or were conducted as natural history studies, i.e. in the absence of any HCV treatment.

Previous HCV treatment was identified as a protective factor whatever the SVR status, which is concordant with results from Butt et al: in patients treated by peg-interferon/ribavirin, all-cause mortality was reduced by 30% to 60% according to treatment duration, when compared with untreated patients, whatever the treatment’s outcome (SVR or not) [36]. This beneficial effect could result from liver fibrosis reduction in patients with no cirrhosis.

Treated-SVR negative patients were at higher risk of all-cause mortality, compared with untreated patients, probably linked to an indication bias (treatment was purposed to patients with bad prognosis). Moreover, no significant reduction of all-cause mortality was found in treated-SVR positive patients contrary to what has been described recently [37]. This could be explained in two ways: 1/ a lack of power, 2/ achieving SVR reduced the overmortality of treated patients, without reaching a protective effect.

The level of CD4+ was not associated with all-cause mortality which is in contrast to what has been found in previous studies including HIV/HCV co-infected patients [20, 37]. This could be explained in three ways: 1/ CD4+ level was a time-dependent covariate which could have modified its association, 2/ the principal causes of mortality in our population are non-HIV and non- liver-related deaths, 3/ most patients had recovered their immunovirological function before inclusion. Nevertheless, the association between CD4+ level and liver-related mortality almost reached statistical significance with an aHR close to the result reported by Grint et al [8].

No association was found between alcohol use and all-cause or liver-related mortality in our study, probably due to the low proportion of excessive alcohol consumers (90 patients, 8.9%). Furthermore, alcohol use was declared by the patient to his physician which might result in an under-reporting (social desirability bias) considering that HCV-infected patients are often aware of the seriousness of excessive alcohol use.

Our study has some limitations. First, the proportion of patients with SVR was low and only few patients were treated by DAA. Even though the SVR rate in our study did not represent the current context of HIV/ HCV co-infected patients, we do not expect that SVR modifies the effect of LSM on all-cause mortality. Indeed, a decrease of LSM values after SVR has been shown, but we would expect that higher LSM markers would still be associated with higher risk of mortality in those with and without SVR. Thus, we do not think that higher SVR rates would have led to different results.

Second, analyses were based on patient records without missing data for variables included in the final models. No imputation procedure was performed because of the small number of patients excluded for missing data and the absence of difference compared to excluded patients. Finally, the main analysis was not adjusted for hepatitis B virus (HBV) status but HBV co-infection was only present in 2.2% of the patients and all were receiving an anti-HBV active cART.

Nonetheless, our study has also several strengths. In the absence of post-SVR cohorts with sufficient follow-up, our approach allows to better understand factors associated with mortality independently of SVR and our primary results were confirmed in several sensitivity analyses. Furthermore, we used an innovative statistical approach to better characterize the association between LSM and all-cause mortality using a joint model. Finally, the interest of our results for clinical practice is important: all HIV/HCV co-infected patients, even those who achieved SVR, have to benefit from a long-term follow-up with LSM to detect patients at high mortality risk.

In conclusion, LSM >12.5 kPa at any point in time was strongly associated with all-cause mortality independently of SVR in HIV/HCV co-infected patients. Close follow-up of these patients should remain a priority even after obtaining SVR. Post-SVR cohorts will be critical in the near future to assess the residual risk especially of liver disease progression and its associated factors. This information is of utmost importance to optimize care especially after the use of DAA.

## Supporting information

S1 Table Factors associated with LSM trajectory in HIV/HCV co-infected patients from the ANRS CO13 HEPAVIH cohort and without missing data (N = 959), longitudinal sub-model of the joint model with shared random effects. (PDF)

## Abbreviations

aHR: adjusted hazard ratio
AIDS: Acquired immune deficiency syndrome
cART: combination antiretroviral therapy
cHR: crude Hazard ratio
DAA: direct acting antivirals
FS: FibroScan
HBV: Hepatitis B virus
HCV: Hepatitis C Virus
HIV: Human Immunodeficiency Virus
HR: Hazard ratio
LSM: Liver stiffness measurement
PCR: Polymerase Chain Reaction
RNA: ribonucleic acid
SVR: sustained virological response
