# Supplementary material for: Increased liver stiffness is associated with mortality in HIV/HCV coinfected subjects: The French nationwide ANRS CO13 HEPAVIH cohort study
Source: PLoS One. 2019 Jan 25;14(1):e0211286. doi: 10.1371/journal.pone.0211286 (PMC6347250; doi:10.1371/journal.pone.0211286)
Supplement: S1 Table — (PDF) [file pone.0211286.s001.pdf]

**S1 Table: Factors associated with LSM trajectory in HIV/HCV co-infected patients from the ANRS CO13 HEPAVIH cohort and without missing data (N=959), longitudinal sub-model of the joint model with shared random effects.**

| | **Adjusted β* [standard-error]** | **p value** |
|---|---|---|
| SVR (t) | | 0.023 |
| -treated-SVR- *versus* untreated | -0.31 [0.13] | |
| -treated-SVR+ *versus* untreated | -0.51 [0.22] | |
| Sex, women *versus* men | -0.27 [0.28] | 0.339 |
| Alcohol : consumption | | 0.548 |
| -past recommended *versus* null | 0.17 [0.41] | |
| -past excessive *versus* null | 0.48 [0.48] | |
| -current recommended *versus* null | -0.02 [0.34] | |
| -current excessive *versus* null | -0.49 [0.51] | |
| CD4+ level (/50cells/mm³) | -0.004 [0.02] | 0.864 |
| Presence of metabolic disorders | 0.07 [0.26] | 0.785 |
| Presence of previous HCV treatment (pretreated) | -0.10 [0.26] | 0.707 |
| Time (age) (/year) | 0.003 [0.01] | 0.829 |
| Sex x time | 0.0001 [0.006] | 0.858 |
| Alcohol x time : consumption | | 0.650 |
| -past recommended *versus* null | -0.001 [0.009] | |
| -past excessive *versus* null | -0.008 [0.01] | |
| -current recommended *versus* null | -0.0009 [0.007] | |
| -current excessive *versus* null | 0.01 [0.01] | |
| CD4+ level x time | -0.0002 [0.0005] | 0.704 |
| Presence of metabolic disorders x time | 0.0007 [0 006] | 0.895 |
| Presence of previous HCV treatment x time | 0.007 [0.006] | 0.246 |

Legend:
* Beta represented change in initial value (or change in slope in case of interaction with time) of ln-transformed LSM (in lnkPa), for one covariate adjusted for the others. For example, for the variable "metabolic disorders": Patients with metabolic disorders had an initial ln-transformed LSM non significantly higher (+0.07 lnkPa) than patients without metabolic disorders, adjusted for the other covariates. Patients with metabolic disorders had a non-significantly higher ln-transformed LSM increase per year (+0.0007 lnkPa) than patients without metabolic disorders, adjusted for the other covariates
